# *In Silico* Docking of HNF-1a Receptor Ligands

**DOI:** 10.1155/2012/705435

**Published:** 2012-12-19

**Authors:** Gumpeny Ramachandra Sridhar, Padmanabhuni Venkata Nageswara Rao, Dowluru SVGK Kaladhar, Tatavarthi Uma Devi, Sali Veeresh Kumar

**Affiliations:** ^1^Endocrine and Diabetes Centre, 15-12-15 Krishnanagar, Visakhapatnam 530 002, India; ^2^Department of Computer Science and Engineering, GITAM University, Visakhapatnam 530045, India; ^3^Department of Biochemistry and Bioinformatics, GITAM University, Visakhapatnam 530045, India; ^4^Department of Computer Science, GITAM University, Visakhapatnam 530045, India

## Abstract

*Background*. HNF-1a is a transcription factor that regulates glucose metabolism by expression in various tissues. *Aim*. To dock potential ligands of HNF-1a using docking software *in silico*. *Methods*. We performed *in silico* studies using HNF-1a protein 2GYP*·*pdb and the following softwares: ISIS/Draw 2.5SP4, ARGUSLAB 4.0.1, and HEX5.1. *Observations*. The docking distances (in angstrom units: 1 angstrom unit (Å) = 0.1 nanometer or 1 × 10^−10^ metres) with ligands in decreasing order are as follows: resveratrol (3.8 Å), aspirin (4.5 Å), stearic acid (4.9 Å), retinol (6.0 Å), nitrazepam (6.8 Å), ibuprofen (7.9 Å), azulfidine (9.0 Å), simvastatin (9.0 Å), elaidic acid (10.1 Å), and oleic acid (11.6 Å). *Conclusion*. HNF-1a domain interacted most closely with resveratrol and aspirin

## 1. Introduction

Hepatic nuclear factor 1 alpha (HNF-1a) is a liver enriched transcription factor that was first discovered in studies aimed at identifying proteins that were responsible for tissue-specific regulation of gene expression in the human liver [1]. These transcription factors were also found in tissues other than liver, including pancreatic islets and kidneys, suggesting they could have a more widespread role in physiological processes [2]. Together, the HNF family is part of a network of transcription factors that together control gene expression during embryogenic development and during adulthood [1]. Genes regulated by HNF-1a also encode products involved in the synthesis of seroproteins, carbohydrates and in detoxification [2]. HNF encoding genes arose by duplication of an ancestral gene at the onset of vertebrate evolution, an evolutionary mechanism for the generation of novel functions [3]. Mutations of HNF transcription family are well known to cause the autosomal dominant maturity onset diabetes of young (MODY), a clinically heterogeneous form of early onset of diabetes resulting from a primary defect in pancreatic beta cell function [4]. Some consider diabetes to be “a disorder of abnormal transcription factors” [4]. However it is now established that MODY results from a dysfunction of transcription factors [5] that regulate beta cell function by controlling downstream targets [6]. Protein-ligand interactions are increasingly employed to derive three dimensional structures of protein complexes. Computational techniques have become important to understand the molecular mechanisms of biological systems, as well as in obtaining leads for therapeutic agent identification. Considering the wide ranging effects of transcription factors in beta cell physiology, and the diverse pharmacological ligands that are available to manage the metabolic disturbances characterized by premature aging of diabetes, we performed an exploratory *in silico* study using various ligands as potential docking partners for HNF-1a.

## 2. Methods

We performed *in silico* studies using HNF-1a protein (PDB id: 2GYP·pdb) and fatty acids (listed in the table) by using the software: ISIS/Draw 2.5 [7], ARGUSLAB4.0.1, HEX5.1 [8].

### 2.1. ISIS/Draw

ISIS/Draw is a chemical drawing GUI software, commonly employed as a chemical structure drawing software following the advent of bioinformatics explosion [7]. It is a simple and concise pure chemical drawing software, which is generally the first choice for use in 2D drawings.

### 2.2. ArgusLab

ArgusLab is a freely distributed software for Windows platform, commonly used as an introductory molecular modeling package especially in the academic environment [8]. It has a user-friendly interface and an intuitive calculation menus. The docking engine approximates an exhaustive search method. It requires a PDB format file for both ligand and receptor [8]. 2D depictions of prospective ligands were drawn using the ISIS/Draw 2.5 and these structures were optimized for energy using AGRUS Lab. The following ligands designed *in silico* were used: resveratrol, aspirin, stearic acid, retinol, nitrazepam, ibuprofen, azulfidine, simvastatin, elaidic acid, and oleic acid.

### 2.3. HEX5.1

The energy optimized ligand structures are docked with HNF-1a using HEX5.1.

## 3. Results

**Figure 1:**
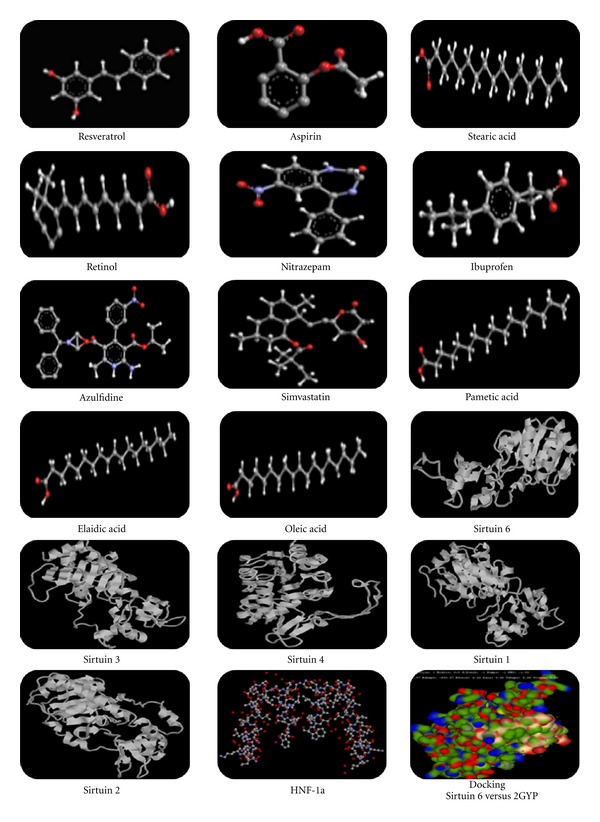
Structures of protein and ligands and docked area.

The docking distances with different ligands are presented in Table 1 and the structures are shown in Figure 1. The decreasing docking distance were as follows: resveratrol (3.8 Å), aspirin (4.5 Å), stearic acid (4.9 Å), retinol (6.0 Å), nitrazepam (6.8 Å), ibuprofen (7.9 Å), azulfidine (9.0 Å), simvastatin (9.0 Å), elaidic acid (10.1 Å), and oleic acid (11.6 Å). 

## 4. Discussion

A convergence of biochemical, mathematical, and computational approaches is being applied to evaluate protein-ligand interactions for identifying pharmacological targets to modulate protein activity. HNF-1a is a transcription factor that belongs to a family of proteins having “DNA binding domains that specifically recognize a short DNA sequence and of an activation or repression domain that influences gene transcription” [5]. It is a conserved protein in vertebrate evolution, composed of three functional domains, belonging to the homeodomain family [4, 5]. It has functions in multiple tissues and organs; an absence of the gene manifests after birth, showing its role in diverse metabolic networks. Recent studies have identified as yet unknown proteins such as the high mobility group protein-B1 (HMGB1) that can interact with HNF-1a [9], forming another layer of regulation in the HNF transcriptional network. Resveratrol activates HNF family of proteins, which exist in all domains of life [10]. Sirtuin resveratrol interactions have led to intense search for compounds that can enhance life-span and delay the process of senescence in tissues and organisms. Sirtuin (Sir-2) was first identified in the bacterium *Saccharomyces cerevisae* as a regulator of DNA recombination, gene silencer, and playing roles in DNA repair and chromosomal longevity. It is believed to be the critical link between caloric restriction and enhanced life span. Ligands of sirtuins, of which resveratrol is the first identified activator, [11], have gained recognition. The only non-genetic and non-pharmacological way to extend life span and prevent the metabolic changes of aging is by calorie restriction: “dietary regimen in which an organism is provided with at least 20% fewer calories than it would naturally consume *ad libitum *while maintaining adequate nutrition” [10]. The effectiveness of resveratrol has been observed *in vitro, in vivo,* and across many species [10]. Considering its effectiveness across species, the underlying mechanism is believed to be “ancient, relatively simple, and well conserved.” Sirtuins, the modulators of this interaction evolved from an ancient molecule that responded to stress and to the availability of food. The family of sirtuins have multiple roles in metabolism, and are modified by dietary changes: SIRT1 promotes fat metabolism in white adipose tissue through interaction with PPAR gamma and adiponectin; it affects pancreatic beta cell in mice by affecting uncoupling protein 2 gene (UCP2) which uncouples oxygen consumption from ATP generation, and dissipates energy as heat; SIRT3 decreases the production of reactive oxygen species; and SIRT4 regulates amino-acid-stimulated insulin secretion in pancreatic beta cells. The sirtuins are versatile energy sensors that enable transcription to sense the metabolic rate of the cell. They act at various levels to repress transcription and to deacetylate non histone proteins including forkhead box type O transcription factor (FOXO) and PPAR gamma [12]. Expressed ubiquitously in human tissues, they show sequence homology and contain conserved catalytic and NAD binding domains. They have important roles in the control of cell proliferation and in metabolic regulation. Together they could be “important determinants of whole-body metabolism and protect against many chronic diseases associated with metabolic dysfunction” [12]. 

Protein docking methods calculate 3D structure of a protein complex starting from its individual structural components, and give information of protein-ligand interactions. Practical difficulties with high throughput structural genomics are likely to result in computational techniques being increasingly employed for understanding biological systems. Undoubtedly protein docking problem is “easy to state but hard to solve.” A number of docking algorithms now use two-step search and score procedures: *ab initio* methods generate an initial list of ligands which are then re-scored with available biophysical information and knowledge-based potentials from analyzing existing interfaces.

HEX5.1 is a commonly used molecular docking package that appropriates an exhaustive search method. The ligand is described as a torsion tree; grids are constructed overlying the binding site. Root node is placed on a search point in the binding site and a set of rotations is created. For each rotation, torsions in breadth-first order are constructed. Those that survive the torsion search are scored. Even though it may not perform as well as commercially available docking methods, HEX5.1 is a first method to narrow lead ligands, owing to its graphical user interface, and its relative speed compared to other complicated algorithms. Even though extending life span without genetic modification and without compromising nutritional status appears inviting, the need for calorie restriction requires volitional control, which is not always easily put into practice. Metabolic networks involving a common set of genes implicated in a switch from Krebs cycle and respiration to glycolysis and glycerol biosynthesis were implicated as a method to extend life span [13]. 

SIRT1 activation has been shown to involve a panoply of processes involving oxidative stress [14], the p53 pathways [15], FOXO [16] as well as DAF 16 [17]. Right from the report of resveratrol activating sirtuins to expand longevity without reducing fecundity [11], a number of compounds were screened for SIRT1 modulation: high-throughput screening was employed to identify compounds with SIRT1 activating and inhibiting potential [18]. Among a library of 147,000 compounds screened, SIRT1 activators had lipolytic and anti-inflammatory properties. Quinoxaline-based potent SIRT1 activators were identified in this study [18]. Similarly, a high throughput *in vitro* fluorescent polarization assay recognized a number of small molecule SIRT1 ligands that were 1,000 times more potent than resveratrol [19]. These bound to SRT1 enzyme-peptide substrate complex at an allosteric site amino terminal to the catalytic domain. In diet-induced obese and in genetically obese mice, these compounds improved insulin sensitivity, lowered plasma glucose, and improved mitochondrial capacity [19]. 

Recent animal studies have shown that chronic supplementation of resveratrol suppressed DNA damage and oxidative stress [20]. Similarly sirtuin 1 antisense oligonucleotide was shown to decrease plasma levels of total cholesterol, by increased cholesterol uptake and export from the liver. This suggested that inhibition of hepatic SirT1 could be a potential method to treat type 2 diabetes mellitus [21]. Along with sirtuins, other pathways and networks may operate in extending lifespan, including forkhead transcription factors and metabolic regulators in mTOR [22]. The “potential longevity pathways are most likely not mutually exclusive.”

In summary our *in silico* docking study suggests that resveratrol and aspirin are ligands that could potentially modulate the hepatic nuclear factor network. It provides a lead for further studies to evaluate such interactions. 

## Figures and Tables

**Table 1:** Docking of ligands with HNF-1a.

Ligand drug/fatty acid	Binding distance with HNF-1a (Angstroms) Å
Resveratrol	3.8
Aspirin	4.5
Stearic acid	4.9
Retinol	6.0
Nitrazepam	6.8
Ibuprofen	7.9
Azulfidine	9.0
Simvastatin	9.0
Palmitic acid	9.8
Elaidic acid	10.1
Oleic acid	11.6
Sirtuin 6	17.6
Sirtuin 3	28.9
Sirtuin 4	43.5
Sirtuin 1	43.5
Sirtuin 2	43.9
Sirtuin 5	43.9
